# Draft Genome Sequence of *Methylomicrobium buryatense* Strain 5G, a Haloalkaline-Tolerant Methanotrophic Bacterium

**DOI:** 10.1128/genomeA.00053-13

**Published:** 2013-06-27

**Authors:** Valentina N. Khmelenina, David A. C. Beck, Christine Munk, Karen Davenport, Hajnalka Daligault, Tracy Erkkila, Lynne Goodwin, Wei Gu, Chien-Chi Lo, Matthew Scholz, Hazuki Teshima, Yan Xu, Patrick Chain, Francoise Bringel, Stéphane Vuilleumier, Alan DiSpirito, Peter Dunfield, Mike S. M. Jetten, Martin G. Klotz, Claudia Knief, J. Colin Murrell, Huub J. M. Op den Camp, Yasuyoshi Sakai, Jeremy Semrau, Mette Svenning, Lisa Y. Stein, Yuri A. Trotsenko, Marina G. Kalyuzhnaya

**Affiliations:** GK Skryabin Institute of Biochemistry and Physiology of Microorganisms, Russian Academy of Sciences, Pushchino, Russiaa; Department of Chemical Engineering, University of Washington, Seattle, Washington, USAb; eScience Institute, University of Washington, Seattle, Washington, USAc; Department of Microbiology, University of Washington, Seattle, Washington, USAd; Los Alamos National Laboratory, Joint Genome Institute, Biosciences Division Genome Science B6, Los Alamos, New Mexico, USAe; Equipe Adaptations et Interactions Microbiennes dans l’Environnement, UMR 7156 UdS–CNRS Génétique Moléculaire, Génomique, Microbiologie, Université de Strasbourg, Strasbourg, Francef; Roy J. Carver Department of Biochemistry, Biophysics and Molecular Biology, Iowa State University, Ames, Iowa, USAg; Department of Biological Sciences, University of Calgary, Calgary, Canadah; Department of Microbiology, Institute of Water and Wetland Research, Radboud University Nijmegen, Nijmegen, the Netherlandsi; Department of Biology, University of North Carolina, Charlotte, North Carolina, USAj; Institute of Crop Science and Resource Conservation–Molecular Biology of the Rhizosphere, University of Bonn, Bonn, Germanyk; School of Environmental Sciences, University of East Anglia, Norwich, United Kingdoml; Division of Applied Life Sciences, Graduate School of Agriculture, Kyoto University, Kyoto, Japanm; Department of Civil & Environmental Engineering, the University of Michigan, Ann Arbor, Michigan, USAn; Department of Arctic and Marine Biology, University of Tromsø, Tromsø, Norwayo; OMeGA, the Organization for Methanotrophic Genome Analysis, Department of Biological Sciences, University of Alberta, Edmonton, Alberta, Canadap

## Abstract

Robust growth of the gammaproteobacterium *Methylomicrobium buryatense* strain 5G on methane makes it an attractive system for CH_4_-based biocatalysis. Here we present a draft genome sequence of the strain that will provide a valuable framework for metabolic engineering of the core pathways for the production of valuable chemicals from methane.

## GENOME ANNOUNCEMENT

Microbial utilization of methane is a key step in the carbon cycle (1–3). Methanotrophs provide an attractive platform for production of commodity chemicals and biofuels from natural gas/renewable biogas (4–6). Over the last 10 years, several novel methane-utilizing microbes have been isolated in pure culture but only a few, including *Methylomicrobium buryatense* strain 5G, show robust growth on methane (5–7).

The draft genome was generated at the Department of Energy (DOE) Joint Genome Institute using Illumina sequencing (8). A short-insert paired-end library (insert size of 270 bp) generated 7.25 Mbp of data (http://www.jgi.doe.gov/). The initial draft data were assembled with Allpaths version 39750 and computationally shredded into 10-kbp overlapping fake reads (9). The initial data were also assembled with Velvet, version 1.1.05 (10), computationally shredded into 1.5-kbp overlapping fake reads, reassembled with Velvet, and shredded into 1.5-kbp overlapping fake reads. The fake reads from the Allpaths and two Velvet assemblies, as well as a subset of the Illumina CLIP paired-end reads, were assembled using parallel Phrap, version 4.24 (High Performance Software, LLC). Possible misassemblies were corrected by manual editing in Consed (11–13). Gap closure was accomplished using repeat resolution software and sequencing of bridging PCR fragments with Sanger and/or PacBio technologies (C. Han, W. Gu, unpublished). Fifty-three PCR PacBio consensus sequences were used to close gaps and to improve the quality of the final sequence. The total estimated size of the genome is 5.4 Mb with an average coverage of 1,343×.

Comparative genome analysis of strain 5G and two other *Methylomicrobium* species, *M. album* strain BG8 and *M. alcaliphilum* strain 20Z, revealed that 5G was most similar to *M. alcaliphilum*, sharing approximately 70% of its proteome at 90% protein sequence identity.

We identified genes encoding membrane-associated methane monooxygenase, soluble methane monooxygenase and an associated chaperon and a transcriptional activator (14), pyrroloquinoline quinone (PQQ)-dependent methanol dehydrogenase, an associated *c*-cytochrome, genes for enzyme assembly and PQQ biosynthesis, tetrahydromethanopterin- and tetrahydrofolate-linked C_1_-transfer pathways, two formate dehydrogenases, and the ribulose monophosphate pathway. The Embden-Meyerhof-Parnas pathway, the Entner–Doudoroff pathway, and the pentose phosphate pathway (transaldolase variant) are predicted. As with the genomes of other gammaproteobacterial methanotrophs, the genome of *M. buryatense* 5G encodes all genes essential for operation of the citric acid cycle and the serine cycle, except for phosphoenolpyruvate carboxylase, isocitrate lyase, and the ethylmalonyl pathway (15,16).

Genes for urea uptake and hydrolysis, assimilatory nitrate/nitrite reduction, dissimilatory nitric oxide reduction, and ammonium uptake were identified. A gene homologous to hydroxylamine oxidoreductase is present (17, 18). The ammonium assimilation inventory includes genes for glutamate and alanine dehydrogenases, glutamate synthase/glutamine synthetase, serine-pyruvate/serine-glyoxylate, and aspartate aminotransferases (19). Genes essential for ectoine biosynthesis were identified.

### Nucleotide sequence accession numbers.

The *Methylomicrobium buryatense* 5G genome sequence was deposited in GenBank/EMBL under the accession numbers AOTL01000000 and KB455575 and KB455576.
